# Phage engineering and phage‐assisted CRISPR‐Cas delivery to combat multidrug‐resistant pathogens

**DOI:** 10.1002/btm2.10381

**Published:** 2022-08-06

**Authors:** Khushal Khambhati, Gargi Bhattacharjee, Nisarg Gohil, Gurneet K. Dhanoa, Antonia P. Sagona, Indra Mani, Nhat Le Bui, Dinh‐Toi Chu, Janardhan Keshav Karapurkar, Su Hwa Jang, Hee Yong Chung, Rupesh Maurya, Khalid J. Alzahrani, Suresh Ramakrishna, Vijai Singh

**Affiliations:** ^1^ Department of Biosciences, School of Science Indrashil University Rajpur Mehsana Gujarat India; ^2^ School of Life Sciences University of Warwick, Gibbet Hill Campus Coventry United Kindgom; ^3^ Department of Microbiology Gargi College, University of Delhi New Delhi India; ^4^ Center for Biomedicine and Community Health International School, Vietnam National University Hanoi Vietnam; ^5^ Faculty of Applied Sciences International School, Vietnam National University Hanoi Vietnam; ^6^ Graduate School of Biomedical Science and Engineering Hanyang University Seoul South Korea; ^7^ Hanyang Biomedical Research Institute Hanyang University Seoul South Korea; ^8^ College of Medicine Hanyang University Seoul South Korea; ^9^ Department of Clinical Laboratories Sciences College of Applied Medical Sciences, Taif University Taif Saudi Arabia

**Keywords:** CRISPR‐Cas9 system, infection, microflora, multidrug resistance, pathogens, phage

## Abstract

Antibiotic resistance ranks among the top threats to humanity. Due to the frequent use of antibiotics, society is facing a high prevalence of multidrug resistant pathogens, which have managed to evolve mechanisms that help them evade the last line of therapeutics. An alternative to antibiotics could involve the use of bacteriophages (phages), which are the natural predators of bacterial cells. In earlier times, phages were implemented as therapeutic agents for a century but were mainly replaced with antibiotics, and considering the menace of antimicrobial resistance, it might again become of interest due to the increasing threat of antibiotic resistance among pathogens. The current understanding of phage biology and clustered regularly interspaced short palindromic repeats (CRISPR) assisted phage genome engineering techniques have facilitated to generate phage variants with unique therapeutic values. In this review, we briefly explain strategies to engineer bacteriophages. Next, we highlight the literature supporting CRISPR‐Cas9‐assisted phage engineering for effective and more specific targeting of bacterial pathogens. Lastly, we discuss techniques that either help to increase the fitness, specificity, or lytic ability of bacteriophages to control an infection.

## 1 INTRODUCTION

In the past few decades, antibiotics have been used extensively for treatment of infectious diseases and preventing food products from spoilage, which has prompted the emergence of antimicrobial resistant (AMR) pathogens. Approximately 0.7 million people die across the globe annually due to AMR pathogens, and it is estimated that this figure could outnumber cancer‐driven mortality by 2050.^1^ In 2017, the global antibiotics resistance market size was USD 7.81 billion, which increased by 5.6% in 2018.^2^ Antibiotic resistance has a direct correlation with antibiotic consumption, and it is predicted that there could be surge of 200% in antibiotic consumption from 2015 to 2030.^3^, ^4^ Antibiotic consumption is relatively higher in low and low‐middle income countries. Furthermore, the drug resistant indices of Pakistan, Vietnam, India, and China are unsettling.^4^, ^5^ A single mutation in a gene can cause an antibiotic to be ineffective for treatment of an infection.^6^, ^7^, ^8^ In addition, horizontal gene transfer is a troublesome strategy among bacteria that leads to aquisition of antibiotic and drug resistance.^9^, ^10^ A pressing need has arisen to treat bacterial infections with antibiotics that possess a novel mode of action or belong to a new chemical class. However, a weak pipeline for antibiotic agents has been reported, and drugs take many years to come to market.^11^, ^12^

Bacteriophages (phages) are viruses that attack specific bacteria and archaea.^13^ Frederick Twort and Felix d'Hérelle independently discovered phages in 1915 and 1917, respectively. In the past, phage therapy was recognized by many leading scientists for eradication of bacterial infections. George Eliava, a Georgian scientist, was one of them. George Eliava and Felix d'Herelle founded the Eliava Institute in Tbilisi, Georgia, in 1923; this institute is dedicated to phage research and phage therapy.^14^ Phage therapy has been used in Russia, Georgia, and Poland.^15^ Similarly, in the United States, Eli Lilly and Company commercialized phage‐based therapy in 1940.^16^, ^17^

Following the discovery of antibiotics,^18^ the use of phage therapy declined worldwide.^19^, ^20^, ^21^, ^22^ However, the number of multidrug resistant (MDR) pathogens has increased, making it difficult to treat them. In the given scenario, phages could be an alternative candidate to treat MDR bacterial pathogens.^23^ With the improved understanding of phage biology, and the availability of its complete genome sequences, genetic tools, genome engineering techniques, and synthetic biology have all led to a resurgence of interest in phage therapy as an antimicrobial strategy.^24^, ^25^, ^26^ The small sizes of phage genomes and the ease with which they propagate within a laboratory set‐up (compared to their eukaryotic counterparts) have ensured that bacteriophages could serve as suitable candidates for generating treatments against bacterial infections.^23^ However, chief limiting factors for application of phages to target MDR pathogens in medical settings include limited host range and the laborious task of phage hunting.^27^ In the context of phage therapies, the host‐range was broadened using a consortium of phages in a single shot, usually referred to as a phage cocktail. This mixed population was tested and held a high degree of efficacy, especially in Eastern Europe.^24^ Ando et al. developed a method for bacteriophage engineering that replaces the viral scaffold to create broad host ranges.^28^ They used an *Escherichia coli* phage for targeting *Yersinia* and *Klebsiella* bacteria by changing the tail fiber and other associated genes.

Recent developments in synthetic phage bioengineering techniques improved the arduous task of phage hunting by effectively tailoring interactions between bacteria and phages. The important factor for successful application of phage bioengineering is efficiency of genome editing. Classical homologous recombineering approaches or more innovative type I‐E clustered regularly interspaced short palindromic repeats (CRISPR) and CRISPR‐associated protein (Cas)‐based counter selection or yeast‐based reconstruction of phage genomes have been useful in the generation of recombinant phages.^28^, ^29^, ^30^ Despite the novel representation of these phage‐bioengineering approaches, successful applications remain elusive, further highlighting the need for a novel approach with higher gene editing efficiency.

One of the promising leads in development of bioengineered phages against MDR pathogens is a CRISPR‐Cas system, which employs a successful genome editing tool in eukaryotic and prokaryotic systems.^31^ These CRISPR systems have been developed and used for target‐specific genome editing in numerous organisms.^32^, ^33^ This technology has overcome the hurdles encountered using gene editing zinc finger nuclease (ZFN) and transcription activator‐like effector nuclease (TALEN) technologies. These difficulties include complex mechanisms, tedious procedures, low‐efficiency, and higher probabilities of off‐target activities.^31^, ^34^, ^35^ CRISPR‐Cas systems are divided into two classes and several subtypes.^36^ Class 1 systems (types I, III, IV) are those with effector complexes using multiple Cas proteins, and Class 2 systems (types II, V, VI) are those that use a single Cas protein.^37^, ^38^, ^39^ The CRISPR‐Cas systems are based on the RNA‐directed endonuclease mechanism that provides adaptive immunity to bacteria against the invading nucleic acids.^40^ DNA sequences based on past encounters, called spacers, are stored in the host chromosome and are used by Cas proteins to detect and cleave invading nucleic acids in cells. This natural system has been engineered into effector complexes expressed from a single plasmid containing Cas protein genes and is used as a guide RNA sequence (gRNA). The gRNA contains spacer and scaffold DNA that allows specifical binding to the complementary target region of gene that is next to the protospacer adjacent motif (PAM) sequence. PAM sites are 2–6 bp in length on the targeted nucleic acid and are required for Cas protein to exhibit its activity. Cas proteins then create a double stranded break (DSB) by cleaving the target nucleotide sequence.^41^ The CRISPR‐Cas system has been successfully employed by various groups to introduce point mutations, reporter gene knock‐in, as well as deletions in various phage genomes.^42^, ^43^, ^44^ In this review, we highlight advances in techniques used for genome manipulation and adaptive evolution to improve the robustness and fitness of phages for therapeutic applications.

## 2 SPECIFICITY OF CRISPR

In the type II CRISPR system, the first 10–12 nucleotides in the 3′ region of the spacer sequence (proximal to the PAM site) are referred as the seed sequence. Mismatch in the seed sequence with the other DNA sequences being targeted would not allow the endonuclease to perform its activity. However, in DNA sequences with close homology, binding might occur, but cleavage is rarely observed. Thus, in the Cas9‐gRNA system, binding the complex to a sequence homologous with the seed sequence could lead to off‐target effects.^45^ Although the effect of off‐target mutations is possibly biased, several attempts have been made to elucidate the factors responsible for Cas9‐gRNA selection specificity.^46^ These factors are broadly divided into two categories: (i) the innate Cas9 specificity that is been encoded in Cas9 endonuclease itself and (ii) the relative availability of the effective Cas9‐gRNA ratio complex relative to the target concentration, with the chance of off‐target effects increasing with higher Cas9‐gRNA concentration.^47^ Thus, for any Cas9 system, one must first predict or explore the profile of off‐target mutations connected to the region of interest. However, the understanding of the molecular mechanism by which Cas9 can occasionally bind to nonspecific regions and cut target sequences is still limited. The fight against this tolerance can be applied to naturally evolved CRISPR as part of the immune “arms race.”^48^

## 3 PHAGE GENOME ENGINEERING AND ASSEMBLY

### 3.1 Phage engineering using homologous recombination

**FIGURE 1:**
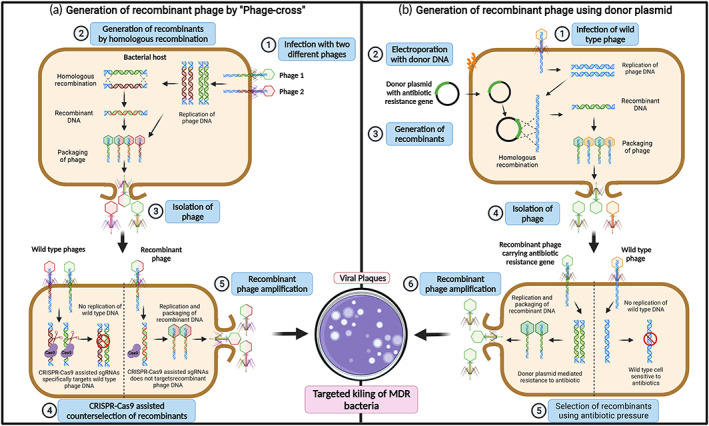
Overview of phage genome engineering and assembly by homologous recombination. (a) Phage engineering to generate mutant phages through classical “phage cross” between two parent phages via homologous recombination. Homologous recombination occurs between homologue DNA sequences enabling introduction target DNA within the phage genome by (1) infecting the bacterial host with two kind of phages. (2) The phage genome can be replicated inside the bacterial host cells to form recombinant phage DNA via homologous recombination between two phage DNA. (3) The wild type and recombinant phages are assembled inside the host cells resulting in lysis of bacterial cells. The lysed phages are isolated and re‐infected into host bacteria. (4) The CRISPR‐Cas9 system‐based counter selection facilitates the removal of wild‐type phages and selection of recombinant phages. (5) The CRISPR‐selected recombinant phage DNA is amplified into bacterial host and is released by cell lysis. The recombinant phage harboring the target gene is used for targeted killing of multidrug resistant (MDR) bacteria. (b) Homologous recombination between wild type phage genome and plasmid DNA. This strategy was developed for generation of recombinant phage particles with genetic mutations, that is, gene insertion, replacement, or deletion using plasmid DNA as an incoming DNA for homologous recombination. (1) The bacterial cells are infected with wild type phage particles and (2) electroporated with the donor plasmid DNA with the targeted genetic mutation along with antibiotic selection marker gene flanked by sequence homologous to the phage genome. (3) The homologous recombination between wild type phage DNA and plasmid DNA results in formation of recombinant phage DNA harboring the desired mutation along with an antibiotic resistance gene. (4) The wild type and recombinant phages are assembled inside the host cells and result in lysis. The lysed phages are isolated and re‐infected into the host bacteria. (5) The recombinant phage particles are selected using selective antibiotic pressure. (6) The antibiotic‐selected recombinant phage DNA is amplified into bacterial hosts and released by cell lysis. The recombinant phages are used for targeted killing of MDR bacteria. Created using BioRender.com.

Engineering a phage genome by homologous recombination within its bacterial host is a well‐established method, and it is one of the most commonly used engineering techniques. Homologous recombination occurs naturally between homologous DNA sequences and enables the introduction of heterologous DNA into the phage genome.^49^ One of the first recombinant phages created was based on homologous recombination of the phenotypes from parental phages by infecting the bacterial host with two kinds of phages. Homologous recombination occurring between genomes helps create a mutant progeny with a mix of phenotypes.^50^ However, use of this method is limited due to the inability to perform site‐specific mutations in the phage genome (Figure 1a).^31^ As a result, the technique was further developed to allow gene introduction by recombination between a phage genome and a plasmid, thereby promoting the use of homologous recombination systems. An insert first must be designed that contains the desired gene flanked by two regions of DNA, which should be homologous to the upstream and downstream sequences of the targeted position in the phage genome.^51^ The insert is cloned into a replicative plasmid and transformed into a host strain for the phage to be engineered. The hosts are then infected with the phage for homologous recombination between the plasmid and the genome. The heterologous gene of interest potentially integrates into the phages genome and will be packed into the progeny phages (Figure 1b).^51^ Finding recombined phages can be difficult and labor intensive, so a reporter gene, such as a fluorescent protein or luciferase, is cloned alongside the gene of interest, which accelerates the identification of mutants by in turn detecting the reporter.^51^, ^52^ Extensive in vitro engineering of plasmids as well as phage genome sequencing are required to verify successful constructs for this technique,^53^ making it a time consuming and potentially difficult process. Furthermore, only a small proportion of phages happen to be recombinant (rates ranging from 10^−4^ to 10^−10^).^51^, ^54^ Due to the low recombination rate, it is unlikely to introduce multiple genes or mutations into the same genome of the desired phage, so when various modifications are needed, they must be made one by one, resulting in a cumbersome process.^49^

### 3.2 Phage recombineering of electroporated DNA

**FIGURE 2:**
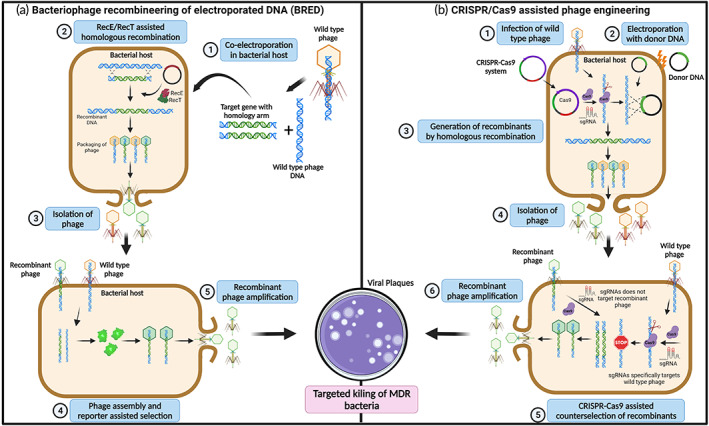
Overview of phage genome engineering and assembly. (a) Bacteriophage recombineering of electroporated DNA (BRED). BRED is based on homologous recombination leveraging RecT and RecE proteins (Rac prophage proteins) to enhance recombination efficiency in electroporated phage DNA. BRED uses donor DNA of interest that is flanked by regions homologues to phage DNA, enabling successful introduction of deletions, exogenous genes, and mutations into the sequence of interest. (1) The wild type phage DNA and targeted gene flanked by phage DNA homology arm are co‐electroporated into host bacterial cells expressing RecE/RecT recombinase system. (2) The homologous recombination between the target gene and phage DNA is assisted by RecE/RecT proteins to generate recombinant phage DNA. (3) The wild type and recombinant phages are assembled inside the host cells, resulting in lysis of bacterial cells. The lysed phages are isolated and re‐infected onto host bacteria. (4) The recombinant phage particles are selected by expression of reporter genes in the host cells. (5) The selected recombinant phage DNA is amplified into a bacterial host and released by cell lysis. The lysed recombinant phages can be employed for various applications including targeted killing of MDR bacteria. (b) CRISPR‐assisted phage engineering for generation of recombinant phages. The CRISPR‐Cas complex expressed within the host specifically identifies and binds to the target site in phage genome and creates DSB, which is lethal to phage replication. However, in the presence of a homologous donor, DSB can be repaired by homologous recombination, generating a recombinant phage. (1) The plasmid encoding all three components of a CRISPR‐Cas system, Cas protein, crRNA, and tracrRNA targeting phage genome, is transformed into a bacterial host infected with wild type phage. (2) The bacteria are then transformed with the donor plasmid DNA harboring gene of interest with mutation along with reporter genes flanked by sequence homology to the phage genome. (3) The CRISPR‐Cas9‐mediated phage genome cleavage is followed by homologous recombination with the donor plasmid DNA, resulting in recombinant phage DNA. (4) The wild type and recombinant phages are assembled inside the host cells, resulting in lysis of bacterial cells. The lysed phages are isolated and re‐infected onto host bacteria. (5) The recombinant phage DNA can be counter selected using a CRISPR‐Cas system by specifically targeting a wild type phage genome and not the recombinant phage DNA. (6) The CRISPR‐selected recombinant phage DNA is amplified into the bacterial host and released by cell lysis. The lysed recombinant phages could be employed for various applications including targeted killing of MDR bacteria. Created using BioRender.com.

Bacteriophage recombineering of electroporated DNA (BRED) is a phage engineering technique first developed by Marinelli et al.^55^ BRED initially was used to modify mycobacteriophages^45^ but has been further developed to modify phages targeting different hosts, such as *Escherichia* and *Salmonella* species.^56^ BRED is based on homologous recombination but additionally uses a RecE/RecT recombination system to enhance the frequency.^31^ The addition of the RecE/RecT system has enabled 10%–15% higher recombination frequencies.^55^ The enhanced version of BRED has various uses, such as gene insertion, deletion, and creating point mutations in phage genomes.^49^, ^57^ The BRED technique uses a DNA substrate, which is the DNA segment of interest, flanked by regions homologous to those upstream and downstream of the phage genome to be modified.^55^ For gene replacements, the DNA substrate needs to contain more than 500 bp of homology, whereas only 45 bp of homology is required for point mutations.^55^ This substrate, along with the phage genomic DNA to be modified, is co‐electroporated into electrocompetent bacterial hosts expressing recombinases; for example, the RecE/RecT‐like proteins from a plasmid.^55^, ^58^ The bacterial cells are then incubated at an appropriate temperature, and any resulting plaques are screened for the probable mutated phage by polymerase chain reaction (PCR) after 24 h (Figure 2a).^55^, ^57^ Although BRED increases recombination frequencies, it comes with the disadvantage of relying on electroporation of both phage and donor DNA. Thus, highly competent bacterial hosts are required for this technique, which limits the method in Gram‐positive bacteria exhibiting low transformation efficiency.^31^ In addition, there is often a high recovery of wild‐type phages in plaques with mutant phages, and therefore extensive PCR screening for recombinant phages is required.^31^

### 3.3 CRISPR‐assisted phage engineering

Recent developments in CRISPR‐Cas‐based genome engineering have significantly improved phage bioengineering. Kiro *et al*. reported the first usage of a type I‐E CRISPR‐Cas system for improved engineering of T7 phage in 2014.^59^ A bacterial host with a plasmid is infected with the phage of interest followed by homologous recombination between the plasmid and phage DNA to delete a gene. Following that CRISPR‐Cas selection can target the gene retained in wild‐type phages from a resulting mixed population to counter select the wild‐type and spare the recombinant phage, which overcomes the time‐consuming process of screening for a recombinant phage in a mixed population (Figure 2b).^49^ Various reports have demonstrated application of the CRISPR‐Cas system from various strains including *Streptococcus thermophiles*, *S. pyogenes*, *Listeria monocytogenes*, and *Staphylococcus epidermidis*. The CRISPR‐Cas system from *S. pyogenes* is most often employed for phage bioengineering as it can efficiently target a wide range of phage genomes to generate recombinant viral progeny.^31^

One of the major rate limiting factors for CRISPR‐Cas‐based phage engineering is the selection of appropriate gRNA.^27^ To overcome this problem, a pipeline has been developed that enables the users to achieve an editing rate >99% for multiple genes in the T4 phage genome. The first and most crucial step in this pipeline is the screening of the most effective gRNA against the gene of interest based on the largest reduction in the efficiency of plating. The study demonstrates the incorporation of the reporter gene nanoLuc luciferase (*nluc*) or *nluc* fused with carbohydrate binding module into *hoc* and *soc* genes of the T4 phage genome.^27^ This was performed by employing a donor plasmid harboring reporter genes flanked by sequence homologous to the target sequence, and expressing the effective gRNA from the same plasmid against the target sequence.^27^ The host must have another plasmid to facilitate Cas9 endonuclease expression. This allows cleavage in the phage genome, followed by homologous recombination with the donor plasmid, enabling the incorporation of reporter gene into the targeted site of phage genome. The successful engineering of T4 phage was demonstrated by a Nano‐Glo luciferase assay.^27^ In the Nano‐Glo luciferase assay, Nano‐Glo luminescent reagent containing furimazine is directly added onto the plaque, which resulted in luminescence due to expressed nanoLuc luciferase enzyme.^60^

In addition to engineering the phage via CRISPR‐Cas technology, strategies have been designed and demonstrated for incorporation of CRISPR‐Cas9 systems into the phage genome for therapeutic applications.^61^ Recently, one such system was devised that allows one‐step incorporation of the CRISPR‐Cas9 system into a lysogenic phage.^62^ The strategy encompasses a suicide vector having an inducible suicide gene *sacB* and a 30‐bp target sequence with a PAM motif. This is introduced into a host along with a prophage and another vector facilitating homologous recombination.^62^ Thereafter, a double stranded donor having a CRISPR‐Cas9 cassette flanked with the sequence homologous to the phage region capable of targeting the 30 bp target sequence in the suicide vector is transformed into the same host.^62^ If the donor is successfully transformed into the cells, CRISPR‐Cas9 could integrate with the prophage, and thereafter the Cas9 endonuclease could be directed toward the 30 bp target sequence and eliminate the suicide vector. If the donor fails to transform into the cell, the cell would be eliminated due to the suicide vector. The said study reported a recombination rate of 28.1%.^62^ The primary advantage of this negative screening strategy is the use of a one‐step recombination step for introducing a CRISPR‐Cas9 cassette in a phage genome without the use of any traditional resistant marker.

Like any genome editing tool, the use of CRISPR‐Cas system has disadvantages. One of the risks is the off‐target activity of the Cas endonuclease; however, this can be overcome by screening through a sequencing approach.^34^ Another potential challenge could be the CRISPR‐Cas inhibitor encoded by the phage. For such phages, CRISPR‐Cas‐mediated genome engineering could be difficult.^35^

### 3.4 Assembly of the phage genome in yeast

**FIGURE 3:**
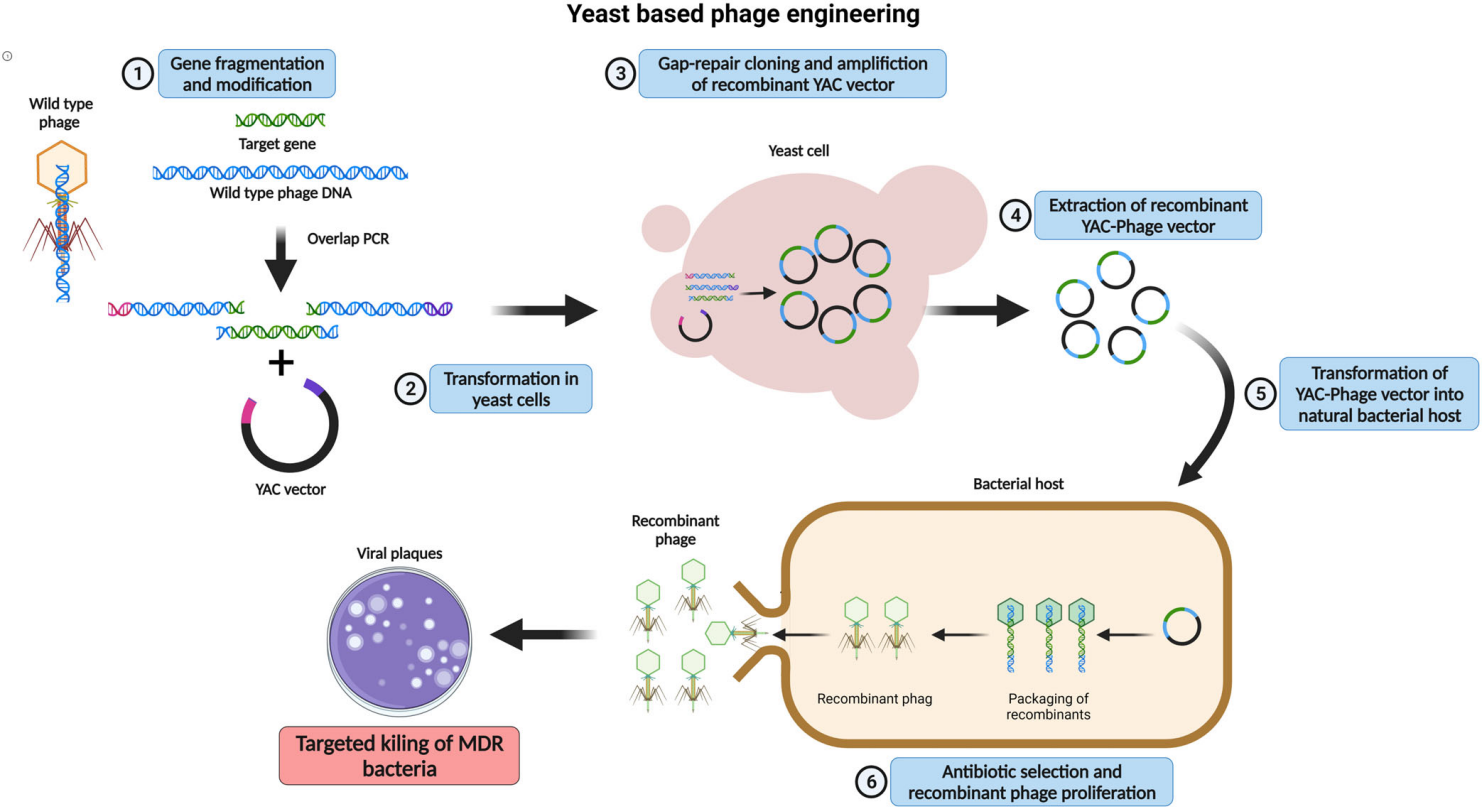
Yeast‐based phage engineering. (1) The polymerase chain reaction purified fragments of phage genome along with fragments harboring gene inserts and reporter gene and a linear yeast artificial chromosome (YAC) vector with overhangs homologous to phage genome are (2) transformed into yeast cells. (3) The transformed phage DNA is assembled into the YAC vector through homologous recombination‐based Gap‐repair cloning. The recombinant YAC vector is amplified inside the yeast cells. (4) The modified YAC‐phage vector is extracted and purified. (5) The purified YAC‐phage vector upon transformation into host bacterial cells generates recombinant phage particles. (6) The resulting recombinant phage particles can be selected using a reporter gene (e.g., antibiotic resistance or fluorescence marker gene). The selected recombinant phage DNA is amplified into the bacterial host and released by cell lysis. The lysed recombinant phages can be used for various applications including targeted killing of MDR bacteria. Created using BioRender.com.

Propagating phage genomes in a bacterial host can be toxic to the host, potentially limiting some engineering methods,^49^ but this could be overcome by using an intermediate cloning host such as *Saccharomyces cerevisiae*. This host has been used to modify the genomes of phages such as K11 and T7.^28^ The use of *S. cerevisiae* eliminates the phage toxicity to allow more stable maintenance of the phage genome and provides more efficient homologous recombination machinery, resulting in higher recombination rates.^63^ During yeast‐based assembly, the phage genome is inserted into a yeast artificial chromosome (YAC) containing overhangs homologous to the phage genome ends that allow them to join by recombination.^63^ The segments are amplified and transformed into yeast, while gap repair joins the fragments to create a modified phage genome within a replicative yeast plasmid. YAC‐phage DNA is then extracted and transformed into a bacterial host to assemble the phage particles (Figure 3).^28^ This technique has been reported to modulate the host range of a phage by modular swapping of phage tail components. As per the report, approximately 25% of clones contained correctly assembled phage genomes.^28^ However, the phage genome needs to be propagated in the bacterial host after engineering, so like BRED, this technique is limited by bacterial transformation efficiencies.

### 3.5 Extending host range

Phages are highly specific toward their bacterial host via tail fibers that attach to specific receptors on the bacterial surface.^64^ This specificity can be exploited to directly target a pathogenic strain of bacteria without impacting the human commensal microbiota.^65^ However, for therapeutic purposes, it is preferable for a phage to have a broader range of possible hosts so that it can be effective against multiple species within a genus and can eliminate the need for a phage cocktail. A phage cocktail is administered if the pathogenic strain is unknown or if multiple variants of the same strain are present. For instance, some Staphylococcal phages can target multiple pathogenic *Staphylococcus* species.^66^ Genetically engineering phages having a broader host range enables faster treatment by eliminating the need to identify the specific strain of pathogen responsible for an infection. In addition, it can also facilitate the drug development process because tracking the pharmacodynamics of multiple phages in one cocktail is complex.^67^ Initial studies have extended the host range of a phage by infecting a host with various phages and selecting phages with mutations, enabling them to propagate in the desired host.^68^, ^69^

A modular engineering approach in yeast has been used for swapping the tail fibers from different phages to enable infection in different hosts.^28^ This is possible because the tail protein of the phage determines the host specificity. In a study, different T7 phage genomes with swapped tail fiber genes from various phage were assembled along with YAC homology ends using Gibson assembly of PCR fragments and were replicated in *S. cerevisiae* cells. The assembled genomes were transformed into *E. coli* cells, producing hybrid T7 phage particles that were able to infect different hosts, such as *Yersinia* and *Klebsiella* strains.^28^ Similarly, another study reported extended host range of phages for DNA transduction into new bacteria by engineering hybrid phage particles expressing multiple tail proteins.^70^ However, unlike previous studies that focused on lytic phages and their ability to propagate, this study used the transducing phage because it was more controllable and likely to receive regulatory approval than virulent phages. Fifteen plasmids were designed, each encoding a gene for a different tail fiber, along with antibiotic resistance cassettes and packing signals.^70^ Plasmids were transformed into host bacteria, and a T7 phage lacking its natural tail genes was able to propagate within the host.^70^ Considering these ample data, this era is replete with literature and novel genome engineering platforms, extending and improving the host range of phage particles, thereby opening gateways for a wide range of DNA transduction or lysis to host cells for therapeutics and other applications.

## 4 PHAGE DISPLAY AND ITS APPLICATION

### 4.1 Phage display

Gregory P. Smith and his partners first introduced phage display by successfully expressing recombinant peptides exposed with capsid proteins of filamentous phages, which constitute the foundation of this powerful tool.^71^ Phage display is an in vitro selection platform applied for protein interaction analysis that uses bacteriophages to connect proteins with their encoding gene. Various bacteriophages such as T4, lambda, and the most popular filamentous phage M13 have been utilized. These bacteriophages can vary in shape or size. However, they must share several important common features such as rapid and natural propagation in bacterial hosts, ability to self‐assemble, and availability of tools for engineering.^72^

In phage display techniques, the DNA encoding interested proteins or peptides is ligated with phage coat‐protein genes, either the minor *pIII* or the major *pVIII* gene, to expose its products on the exterior of the phage coat while the DNA remains inside. The preparation of phage display libraries is the first step in the procedure and is known as “biopanning.” By immobilizing the target proteins on the surface of a plate or bead, phages that display the protein that binds to one of those targets remain bound after washing the plate, while others are removed.^73^ A combination of low‐pH elution buffer and sonication is then used to weaken the linkage between proteins and targets while conserving attachment of the target molecule to the surface.^74^ Thus, the binding phages are eluted and amplified by bacterial infection, usually with *E. coli* strains, to enrich the pool of specifically binding phages. After several cycles, the corresponding DNA is sequenced to identify the target proteins.^73^, ^75^

The phage display technique focuses on building a library of peptides or antibody variants, which are then selected based on their binding characteristics.^76^ Phage display can help provide a variety of phage clone groups, each expressing a random sequence. Phage display has advantages over other methods for library screening. First, while the number of plaques or colonies screened by hybridization in the standard screening of cDNA is limited, phage display can screen a large number of clones at once. Second, this technique can establish a physical linkage between the phenotype, which is the protein of interest displayed outside the phage coat, and the genotype (the DNA sequence encoding this protein) within the same viral particle. Finally, phage display can produce diversified libraries of proteins contemporarily exposed on the surface of the phage coat.^73^

### 4.2 Application of phage display

Phage display is essential in the field of bioengineering and immunology and is also used for development of vaccines and drug molecules.^76^, ^77^ One of the outstanding advantages of phage display over other nanotechnology platforms is that it provides rapid and uniform replication for sustainable and cost‐effective production on a larger scale.^78^

An important step of vaccine development is to select the appropriate antigen and adjuvant. Phage‐based vaccination is achieved by exposing multiple foreign antigen copies on the capsid surface of immunogenic particles to obtain an effective immune response. The combinatorial peptide libraries of the phage display techniques also help to identify promising vaccine candidates against different diseases, particularly against microbial infections. Based on an affinity selection strategy, these libraries are screened to select mimotopes with high antigenicity and immunogenicity.^79^

Cancer immunotherapy is considered a promising approach to treat cancer as an alternative to or in addition to chemotherapy and radiation because of its ability to minimize systematic side effects.^80^ Current experiments on animal models are focused on several concepts of this anti‐cancer immune response, and the phage display platform is modified to match each concept. In one such concept, tumor‐associated antigens (TAAs) are considered as rational targets for cancer treatment. Thus, the biopanning process of phage display toward monoclonal as well as polyclonal antibodies (Abs) could be performed to detect TAA mimotopes.^81^, ^82^ A better solution is to produce anti‐TAA Abs continuously and endogenously in a process called active vaccination. It aims both to eliminate cancer and to decrease the risk of recurrence.^83^ In this case, utilization of phages as vaccine carriers stimulates both CD4^+^ and CD8^+^ T lymphocytes and induces strong cytotoxic responses.^84^, ^85^ The goal of active vaccination is to use peptides derived from phage display to indirectly promote the immune response via its checkpoint inhibitors or to modulate immune cell activity.^72^

In the field of infectious diseases, an ideal treatment of an infection involves a specifically targeted antibiotic to kill the pathogen instead of broad‐spectrum ones, as broad‐spectrum antibiotics could lead to antibiotic resistance. McCarthy and partners constructed a phage library incorporating *N*‐acryloyl‐3‐aminophenylboronic acid moieties to expose dynamic covalent binding to the surface of bacterial cells.^86^ This library was screened against live bacterial cells to yield a potent and selective binder of *Acinetobacter baumannii* and *S. aureus*. Thus, this phage display platform supports rapid identification of peptide probes of the specific pathogen converted into bactericidal agents with high specificity.^86^ Furthermore, there are several successful drug discovery stories based on phage display techniques. Peptide‐based therapeutics are examples of such drugs that have come to market. In addition, the clinical usage of peptide‐based therapeutics is predicted to increase over the next few years, and phage display is expected to provide lead molecules for further screening to generate an arsenal of therapeutic compounds.^73^ With the advancements in phage engineering and genome sequencing techniques, phage display can be exploited for the betterment of humankind. Moreover, due to recent progress in developing tools, phage engineering, including synthetic phage engineering, phage protein engineering, and phage‐inspired antibacterial design, has re‐emerged as a potential approach for eliminating AMR bacteria.^87^

## 5 CRISPR‐Cas9‐BASED PHAGE DESIGN FOR CONTROLLING BACTERIA

As a proof of principle, the use of CRISPR‐Cas9 alone or in combination with phages for eradication of several MDR pathogens has been successfully shown in multiple studies. A brief description of such studies is listed in Tables 1 and 2.

**TABLE 1:** CRISPR‐Cas‐based phage design for controlling bacteria.

Phage	Target organism		Genome engineering in phage	Genes targeted in host organism	In vivo model	Highlights	References
	Name	Description					
ΦNM1	*S. aureus* RNK	Kanamycin‐resistant	Phage loaded with pDB121::aph, pDB121::mecA	*aph‐3*, *mecA*	CD‐1 mice	Antibiotic resensitization Growth inhibition Selective killing Prevention of skin infection in mice model	^91^
	*S. aureus* USA300	Methicillin‐ and tetracycline‐resistant					
M13	*E. coli* EMG2 pNDM‐1	β‐Lactam resistant	Phage loaded with RGN*ndm‐1*, RGN*ndm‐1/shv‐18*, RGN*eae*	*bla*_NDM‐1_, *bla*_SHV‐18_, *gyr*_AD87G_, *eae*	*G. mellonella*	Growth inhibition Multiplexing against different genetic signatures Targeting point mutant protein with antibiotic‐ resistance Remodulating synthetic consortium	^90^
	*E. coli* pSHV‐18	β‐Lactam resistant					
	*E. coli* O157:H7	Enterohemorrhagic strain					
	*E. coli gyr*_AD87G_	Quinolone resistant					
	*E. coli* CJ236	Chloramphenicol resistant					
T7	*E. coli* DS7045	*E. coli* BL21A carrying/pWUR397/pWUR400/pAnti‐1.7	1.7 gene, 4.3 gene	NA	NA	A simple and efficient method to genetically engineer the *E. coli* phage T7 using *E. coli* type I‐E CRISPR‐Cas system.	^59^
	*E. coli* RK6617	*E. coli* BL21A carrying/pWUR397/pWUR400/pAnti‐4.3					
λ prophage	*E. coli* pNDM, or pCTX	Streptomycin resistance	CRISPR cascade genes and array in phage genome^a^	*ndm‐1*, *ctx‐M‐15*	NA	A strategy to sensitize bacteria to antibiotics and selectively kill antibiotic resistant bacteria	^92^
φSaBov	*S. aureus* CTH96	Bovine isolate susceptible to ϕSaBov	Programmed CRISPR‐Cas9 system in noncoding region of phage genome^b^	*nuc*, *esxA*	C57BL/6 mice	Growth inhibition of *S. aureus* Complementation of tail fiber for phage broad host range Removal of toxic gene from host chromosomes Decolonization of *S. aureus* from skin of mouse model	^148^
	*S. aureus* ST1, ST5, ST8 and ST36	Human pandemic clonal lineage					
phiKpS2	*K. pneumoniae* S2	Mutant of *K. pneumoniae* DSM 2026	Δgp1_8 and Δholin	NA	NA	A CRISPR driven strategy for precise genome engineering in phage	^43^
φSaBov	*S. aureus* ATCC 6538‐GFP	A human isolated *S. aureus* strain engineered to express GFP	Programmed CRISPR‐Cas9 system in noncoding region of phage genome^b^	*nuc*	Sprague Dawley female rats	Mitigating biofilm forming *S. aureus* infection In vivo rat model for osteomyelitis and soft tissue infection Delivery of phage or phage‐fosfomycin therapeutics with alginate hydrogel Phage reducing soft tissue infection but not bone infection	^149^
M13	*E. coli* NEB5‐α *bla*_*IMP‐1*_ */bla*_*OXA‐48*_ */bla*_*VIM‐2*_ */bla*_*NDM‐1*_ */bla*_*KPC‐2/mcr‐1/mcr‐2*_	Carbapenem‐or colistin‐resistant	EC‐CapsidCas13a_blaIMP‐1/1/blaOXA‐48/blaVIM‐2/blaNDM‐1/blaKPC‐2/mcr‐1/mcr‐2, SA‐CapsidCas13a_mecA, *mcr‐1*, *mcr‐2*	*bla*_IMP‐1_, *bla*_*OX48*_, *bla*_*VIM‐2*_, *bla*_*NDM‐1*_, *bla*_*KPC‐2*_, *mecA*	*G. mellonella*	Sequence‐specific bactericidal activity Sequence‐specific killing to modulate the complex microbial flora In vivo therapeutic effect in *G. mellonella* infection model	^94^
	*S. aureus* USA300	Methicillin‐resistant					
M13	*E. coli* Sm^R^ F+ sfgfp	Streptomycin resistant, green fluorescence protein expression	Phage loaded with pCas9‐GFPT‐f1A/B	*sfgfp*	BALB/c mice	Oral administration of M19 phage loaded with CRISPR‐Cas9 expressing phagemid for targeting sequence specific gene in gut microbiome A proof of principle for in vivo targeting strain specific organisms in gut microbiome	^93^
	*E. coli* Sm^R^ F+ mcherry	Streptomycin resistant, red florescence protein expression					
vB_EcoM‐IME365	*E. coli* MG1655 pUCtarget_k_	Kanamycin‐resistance	Phage genome integrated with CRISPR‐Cas9 cassette from pCas9 plasmid	*bla*_NDM‐1_	BALB/c mice	Phage‐delivered resistance eradication with subsequent antibiotic treatment (PRESA) strategy to combat drug resistant pathogen The designed strategy displayed superior antimicrobial activity compared to lytic phage alone in in vitro and in vivo mouse skin and intestinal infection model	^62^

^a^ CRISPR cascade genes: *cse1*, *cse2*, *cas7*, *cas5*, *cas6e*, *and cas3* of *E. coli* type I‐E CRISPR system, and CRISPR array encoding spacers to targeting *ndm‐1* and *ctx‐M‐15*.

^b^ CRISPR‐Cas9 system: tracrRNA, crRNA, and spCas9.

pDB91: a phagemid system containing *rinA*, *ter*, *S* and *terL* genes and packaging site of ΦNM1; pDB121::aph: pDB91 carrying spCas9, tracrRNA, and minimal CRISPR array to target *aph‐3*; *Aph‐3*: aminoglycoside phosphotransferase gene; *mecA*: penicillin binding protein 2a; *bla*_*ndm‐1*_: New Delhi metallo‐β‐lactamase‐1; *bla*_*shv‐18*_: β‐lactamase; *gyr*: gyrase; *eae*: intimin; 1.7: nucleotide kinase; 4.3: Gp4.3; *ctx‐M‐15*: Cefotaximase‐Munich; *mcr‐1*: probable phosphatidylethanolamine transferase; *nuc*: micrococcal nuclease; *esxA*: Type VII secretion system extracellular protein A; *bla*_IMP‐1_: β‐lactamase; *bla*_*OXA‐48*_: β‐lactamase, *bla*_*VIM‐I*_: β‐lactamase class B VIM‐2: *bla*_*KPC‐2*_: β‐lactamase; *sfgfp*: super folder green florescence protein; *mecA*; pDB121::mecA: pDB91 carrying spCas9, tracrRNA, and minimal CRISPR array to target *mecA*; RGN: a phagemid system containing f1 origin and RNA guided nuclease construct; RGN*ndm‐1*: RGN targeting New‐Delhi metallo‐β‐lactamase 1 gene; RGN*ndm‐1/shv‐18*: RGN targeting both *ndm‐1* and *shv‐18* genes; RGN*eae*: RGN targeting both *eae* genes; pWUR397: *cas3* under T7 promoter, KanR; pWUR400: cascade genes under T7 promoter, StrR; pAnti‐1.7 pWUR477 cloned with anti‐1.7 spacer; pAnti‐4.3 pWUR477 cloned with anti‐4.3 spacer; pCas9‐GFPT‐f1A/B: GFP targeting CRISPR‐Cas9 phagemid with *bla* and f1 ori sequence; EC‐Capsid‐Cas13a_blaIMP‐1/1/blaOXA‐48/blaVIM‐2/blaNDM‐1/blaKPC‐2/mcr‐1/mcr‐2: Vector expressing Cas13a and spacers for targeting either *bla*_IMP‐1_ */bla*_OXA‐48_ */bla*_VIM‐2_ */bla*_NDM‐1_ */bla*_KPC‐2/*mcr‐1/mcr‐2*_, respectively; SA‐CapsidCas13a_mecA: vector expressing Cas13a and spacer for targeting *mecA* in *S. aureus*_._ pUCtarget_k_: pUC plasmid containing kanamycin resistance marker with 5′‐end consisting PAM sequence from *blaNDM*.

**TABLE 2:** Proof of concept for CRISPR‐Cas‐assisted antimicrobial strategy.

Target organism		Gene targeted in host organism	Cas protein	Highlights	Ref
Name	Description				
*E. coli* CFT073	Uropathogen	*papG*	CQD‐Cas9	Carbon quantum dot‐mediated delivery of Cas9 and gRNA for targeting fimbria adhesion gene Efficient reduction in adhesion and biofilm formation Increased survival of *Caenorhabditis elegans* when treated with CDQ‐Cas9 during infection study	^100^
*E. coli* C600	Colistin resistant	*mcr‐1*	spCas9	In vitro elimination of *mcr‐1* harboring plasmid	^98^
*K. pneumoniae* 51933	Carbapenem resistant	*bla*_*KPC*_, *bla*_*NDM*_, and *bla*_*OXA‐48*_, *repA*, *repB*, *parA*	spCas9	Curing of carbapenemase genes and plasmid in several *Enterobacteriaceae*	^114^
*K. pneumoniae* 13001					
*K. pneumoniae* Kp97_58					
*K. pneumoniae* 49210					
*S. marcescens* SmN01					
*E. coli* 28009					
*E. coli* 53433					
*E. xiangfangensis* 34399					
*E. hormaechei* 34978					
*E. coli* 14EC033	MDR isolates from the fecal samples of patients with diarrheal diseases	*nikA*, *hicB*, *mcr‐1*, *vagC*, *sopA*	spCas9	CRISPR‐Cas9 system for in vitro curing of *mcr‐1* harboring plasmid	^97^
*E. coli* 14EC007					
*M. smegmatis* mc^2^ 155	Derivative of *M. smegmatis* mc(2)154 and susceptible to kanamycin	NA	Cas1	Altered stress response and impaired DNA damage repair in Cas1 recombinant strain. Cas1 increases the susceptibility against multiple antitubercular agents	^122^
*K. pneumoniae* 5573	Clinically isolated strain	*fosA*, *bla*_*KPC‐2*_, *bla*_*SHV*_, *bla*_*CTX‐M‐65*_	spCas9, nSpCas9‐APOBEC1	Precise base conversion using a fusion protein of cytidine deaminase and Cas9 nickase Highly efficient genome editing system by harnessing CRISPR‐Cas9 and lambda Red recombination system Enhancing the susceptibility of *K. pneumoniae* toward fosfomycin by disrupting or creating a premature stop codon in the *fosA* gene Inactivation of carbapenem resistant genes and deletion of ESBL genes	^113^
*K. pneumoniae* KP_CRE23	Hypermucoviscous carbapenem‐resistant				
*E. coli* NJ‐15‐3	Colistin resistant	*mcr‐1*	spCas9	Colistin resensitization of *E. coli* by targeting *mcr‐1* Bovine myeloid antimicrobial peptide‐27‐1 enhanced pCas plasmid delivery into bacterial cells	^96^
*E. coli* BW25113	Ampicillin and ceftazidime resistant	ESBL‐TMV ESBL‐SHV	spCas9	Strain resensitization by targeting conserved sequence shared among >1000 ESBL	^95^

ESBLs: extended‐spectrum β‐lactamases; spCas9: *S. pyogenes* Cas9; *mcr‐1*: probable phosphatidylethanolamine transferase; *nikA*: NikA; *hicB*: antitoxin HicB; *vagC*: virulence‐associated protein VagC; *sopA*: plasmid partition protein SopA; *papG*: fimbrial adhesion; CDQ‐Cas9: carbon quantum dots conjugated Cas9; *fosA*: Mn^2+^‐ and K^+^‐dependent glutathione *S*‐transferase; *bla*_*KPC‐2*_: β‐lactamase; *bla*_*shv*_: β‐lactamase; *bla*_*CTX‐M‐65*_: β‐lactamase Cefotaximase‐Munich; nSpCas9‐APOBEC1: Cas9 nickase conjugated with the murine cytidine deaminase rAPOBEC1; *bla*_*NDM*:_ New Delhi metallo‐β‐lactamase; *bla*_*OXA‐48*_: β‐lactamase; *repA*: IncFIIK replication gene repA; *repB*: IncFIB replication gene B; *parA*: plasmid partition gene A; Cas1: CRISPR‐associated endonuclease Cas1.

### 5.1 Escherichia coli

**FIGURE 4:**
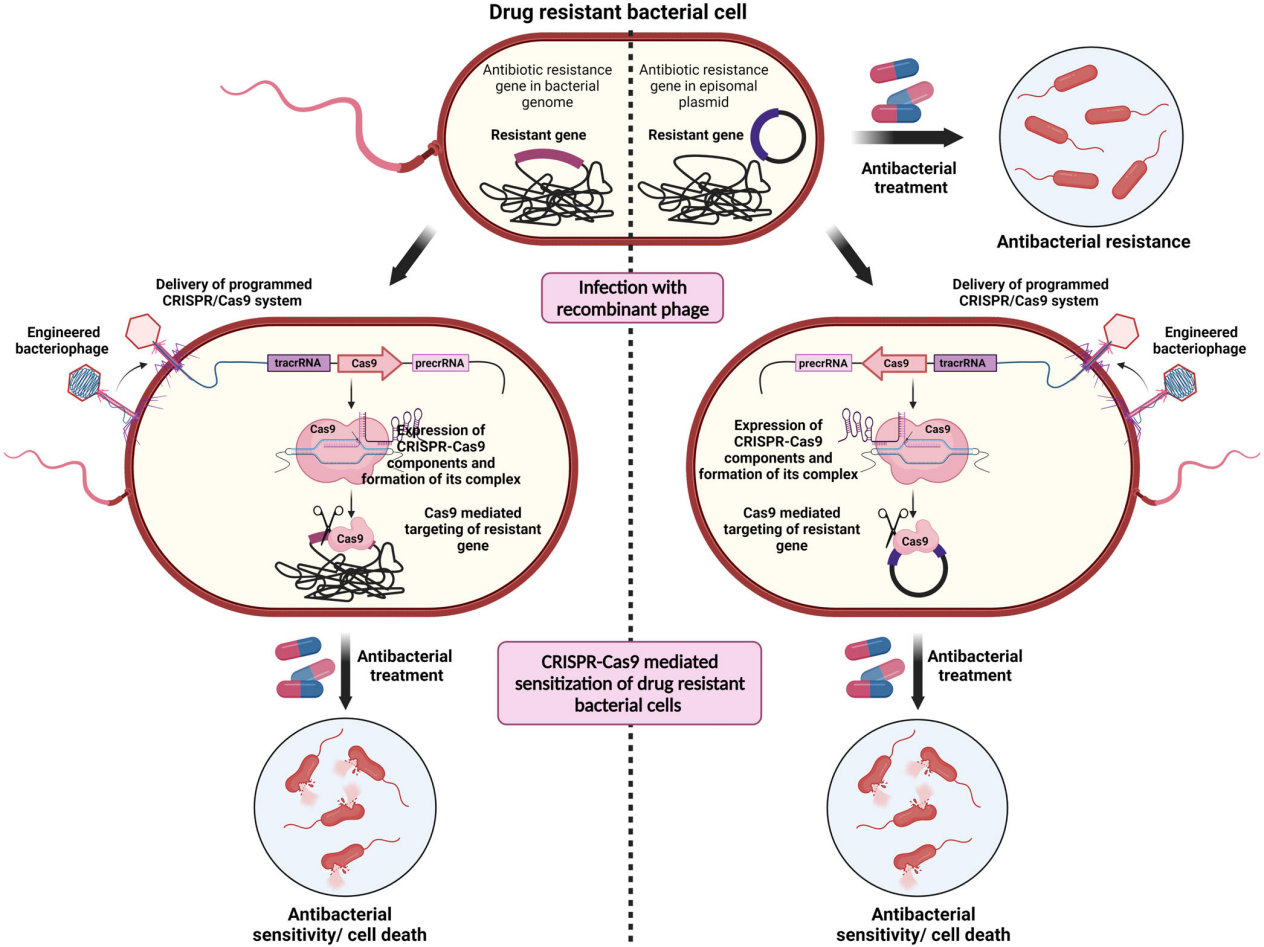
CRISPR‐Cas system against MDR bacteria. The resistance gene can be carried on a chromosome or/and a plasmid conferring resistance toward antibacterial treatment. The CRISRPR‐Cas9 system has been used as an effective antibacterial agent. The antibacterial resistant bacterial cells are transduced with recombinant bacteriophage carrying the Cas9 nuclease along with guide RNA (gRNA) against target sequences in plasmids or chromosomes. The CRISPR‐Cas9 system identifies and cleaves target resistance gene sequences on a chromosome or plasmid, leading to resensitization of MDR bacterial cells to antibiotics and eventual bacterial cell death. Created using BioRender.com.

*E. coli* is a well‐known prokaryotic model organism that is frequently used for the study of genetics, physiology, metabolism, and biochemistry. Due to its readily available molecular biology toolbox, it has emerged as a bacterium that is easy to manipulate. However, this gut symbiont is not only a “laboratory workhorse,” but is also known to cause intra‐intestinal and extra‐intestinal diseases such as bacteremia and urinary tract infection (UTI).^88^ World Health Organization (WHO) has also enlisted the said pathogen with carbapenem‐ or cephalosporin‐resistant profiles as critical for antimicrobial development. In addition, Asia and Europe have high prevalence of colistin resistance among clinically isolated *E. coli*. This is concerning because colistin is considered the last line of antibiotic treatment.^89^ To treat an MDR bacterial pathogen exhibiting resistance to the last line of antibiotics, a novel approach could be to use phages, which are natural killers of their respective bacterial cell. Furthermore, the discovery of the CRISPR‐Cas9 system has enabled users to resensitize the pathogen against antibacterials using bacteriophages as delivery agents (Figure 4). In 2014, two papers were published in *Nature Biotechnology* ^90^, ^91^ on the development of CRISPR‐Cas9 technology for phage‐mediated sequence‐specific targeting and removal of bacterial pathogens, wherein *E. coli* and other microorganisms were used for experimental demonstrations.

In a quest for a new therapeutic strategy against MDR pathogens, a study demonstrated that M13 bacteriophage loaded with phagemid facilitating CRISPR‐Cas9 expression that targets genes such as New Delhi metallo‐β‐lactamase‐1 (*bla*_*ndm‐1*_), β‐lactamase (*bla*_*shv‐18*_), intimin (*eve*), and DNA gyrase A (*gyrA*_D87G_) could result in MDR *E. coli* growth inhibition.^90^ CRISPR‐Cas9 system was used for chromosomal targeting of genes that led to a 1000‐fold decrease in transformation efficiency compared to the wild‐type strain. The developed platform also enabled sequence‐specific multiplexing against different genetic signatures of target organisms.^90^ The system reported was sensitive enough to recognize and target the point mutant gene *gyrA*_D87G_ confering quinolone resistance. The same study also investigated a phage system enabling the user to modulate the bacterial population in the consortium by selectively knocking down the genetic signature of the targeted strain using CRISPR‐Cas technology.^90^ Similarly, phage‐delivered resistance eradication with a subsequent antibiotic treatment (PRESA) strategy has been proven to eradicate kanamycin‐resistant *E. coli* in in vitro and in vivo mouse skin and intestinal infection models. The lysogeny phage equipped with the CRISPR‐Cas9 cassette in the genome was used for sensitization of drug‐resistant bacterial cells in a PRESA strategy.^62^ As per the report, the PRESA strategy enabled 6‐ and 5‐log reduction in bacterial load in vitro and in vivo, respectively. Interestingly, the bacterial cells acquired resistance against lytic phages within 24 and 48 h in in vitro and in vivo mouse model, respectively, however, the use of the PRESA strategy had a constant effect, and no resistant mutants were observed.^62^ In another study, CRISPR‐integrated temperate and lytic bacteriophage λ were designed for sensitization as well as killing of antibiotic‐resistant *E. coli* to control the infection.^92^ A genome engineered phage expressing CRISPR cascade genes of *E. coli* type I‐E CRISPR system and CRISPR array sequence, together targeting *ndm‐1* and Cefotaximase‐Munich (*ctx‐M‐15*), resulted in removal of the plasmid‐based resistant bacteria but maintained the sensitive bacteria.^92^ This study created a foundation for using an engineered phage to clean hospital surface areas without harming the sensitive bacteria.^92^ Recently, bacteriophages have also been used to deliver a CRISPR‐Cas9 system in the gut microbiome of mice for strain‐specific genomic deletions. In this work, the authors demonstrated the possibility of generating genomic deletions in *E. coli* by targeting GFP via oral administration of M13 bacteriophage loaded with phagemid facilitating CRISPR‐Cas9 expression.^93^ The study provides a model for understanding as well as a proof of principle for in vivo strain‐specific targeting, facilitating clinical application of phage‐based CRISPR‐Cas therapeutics.^93^ Apart from Cas9 nuclease, CRISPR‐Cas13a has also been used to target specific organisms using bacteriophages as a delivery agent.^94^ A construct that enabled Cas13a and spacer expression was packed in an M13 capsid. This helped to target different carbapenem‐, colistin‐, and methicillin‐resistant genes in *E. coli* and *S. aureus* that resulted in 2‐ to 3‐fold greater bactericidal activity compared to that of Cas9 nuclease. The same system also demonstrated in vivo therapeutic effects when tested in *Galleria mellonella* as a model organism.^94^

Many similar studies have been conducted where CRISPR‐Cas9 was used to resensitize *E. coli* against antibiotics. For example, a CRISPR‐Cas9 system was developed for knocking out the antibiotic‐resistant gene to resensitize *E. coli* that were further targeted with antibiotics for growth inhibition and control of infections. The system was designed in a manner that it targets a conserved sequence shared among >1000 extended‐spectrum β‐lactamase mutants.^95^ In another study, a “superbug” gene *mcr‐1*, which was discovered in China (2016), was efficiently knocked out in *E. coli* using the CRISPR‐Cas9 system, and proven to be an important anti‐resistant strategy.^96^ Similarly, resensitization of *E. coli* to colistin using a CRISPR‐Cas9 system by targeting *mcr‐1* in plasmids has been reported in several other studies.^97^, ^98^ Recently, a CRISPR interference (CRISPRi) system was used for targeting class I integrons in *E. coli* for reduction of MDR; here, Class I integrons are primarily responsible for AMR transfer among bacterial cells.^99^ CRISPR‐Cas9 systems have also proved efficient for reducing adhesion and biofilm formation in uropathogenic *E. coli* by targeting virulence factors such as fimbrial adhesion (*papG*).^100^ All of the aforementioned studies hold high possibility to be further expanded to other genes of *E. coli* using CRISPR‐Cas9 for efficient control and management of infections. Phage is an option for packaging of CRISPR‐Cas9 tool for specific delivery in bacterial pathogens and removal of the same from a mixed population.

### 5.2 Klebsiella pneumoniae

*K. pneumoniae* is an opportunistic pathogen that causes a wide range of nosocomial infections such as pulmonary pneumonia, UTIs, bacteremia, meningitis, and liver abscesses.^101^, ^102^, ^103^, ^104^ A meta‐analysis showed a pooled mortality of 42.14% among patients infected with carbapenem‐resistant *K. pneumoniae* (CRKP).^105^ The risk of mortality is even higher if the CRKP infection is associated with the bloodstream, in patients admitted to the ICU, or if an individual has undergone a solid organ transplant. Geographically, people in Europe, followed by Asia suffer the highest mortality when infected with CRKP compared to its susceptible variant.^105^ The Center for Disease Control and Prevention (CDC) in 2013 and the WHO^106^ have listed *Enterobacteriaceae* with carbapenem‐ or cephalosporin‐resistant profiles (including *K. pneumoniae*) among critical pathogens requiring urgent action. In addition, a pressing need has arisen to control and eradicate *K. pneumoniae*, exhibiting resistance towards several antibiotics including colistin.^107^, ^108^ Antibiotic‐resistant *K. pneumoniae* has become a serious problem in clinics and requires immediate measures.^109^

Phages have the potential to replace antibiotics and have recently attracted much scientific and public attention for treating MDR bacterial infections.^110^ For instance, a bacteriophage TSK1 belonging to the *Siphoviridae* family is capable of both reducing *K. pneumoniae* growth and efficiently reducing *K. pneumoniae* biomass in biofilms during its post‐ or pre‐treatment. This phage also happens to be stable at 37 °C at pH 7. Likewise, bacteriophage SU503, SU552A, ϕKp16, and ϕKp27, and bacteriophage ϕKp34 and ϕKp24 belonging to *Autographiviridae* and *Myoviridae* families, respectively, have been characterized and are found to be able to kill clinical isolates of *K. pneumoniae*.^111^, ^112^ In addition, a CRISPR‐Cas9‐based efficient and cost‐effective procedure for genome editing in *K. pneumoniae* bacteriophage phiKpS2 has been reported.^43^ A homologous region in the phiKpS2 (30–60 bp) has been identified for creating mutations, gene deletion, and swapping. The study also involved a frameshift mutation to verify essential and nonessential genes in the phage genome.^43^ The authors also successfully deleted a putative promoter and nine genes of phiKpS2 using the CRISPR‐Cas9 technology. Holin was also deleted, which interestingly lead to a slight effect on phiKpS2 infection. This finding could facilitate CRISPR‐based phage engineering for future phage therapy to treat *K. pneumoniae* infections.^43^ Even though phages for targeting *K. pneumoniae* have been characterized and its genome engineering strategy has been developed, the use of phages for delivering CRISPR‐Cas9 vectors to target *K. pneumoniae* is a missing piece in the literature. However, several studies have reported the use of CRISPR‐Cas9 systems to target MDR *K. pneumoniae*. For example, Wang et al. designed a plasmid expressing CRISPR‐Cas9 for targeting the fosfomycin resistance gene (*fosA*) in *K. pneumoniae* 5573 that enhanced their susceptibility toward fosfomycin. Two plasmids, namely, pCasKP and pSGKP were used wherein, the former facilitated CRISPR‐Cas9 expression, while the latter enabled λ Red recombination. When used together, the system results in highly efficient DSBs. In the same study, Cas9 nickase conjugated with the murine cytidine deaminase rAPOBEC1 was used to create premature stop codons in the *fosA* gene of *K. pneumoniae* 5573.^113^ In a similar manner, both previously mentioned strategies have also been used to resensitize hypermucoviscous *K. pneumoniae* KP_CRE23 to carbapenems by targeting the carbapenemase gene, *bla*_*KPC*‐2_, and two of the extended spectrum β‐lactamases genes, *bla*_*SHV*_ and *bla*_*CTX‐M‐65*_.^113^ Likewise, CRISPR‐Cas9 has been used for resensitization of carbapenem‐resistant *Enterobacteriaceae* (CRE) to carbapenems. Genes such as *bla*_*KPC*_, *bla*_*NDM*_, and *bla*_*OXA‐48*_ within the clinical isolates of carbapenem‐resistance *K. pneumoniae*, *E. coli*, *Enterobacter hormaechei*, *E. xiangfangensis*, and *Serratia marcescens* have been targeted, leading to a promising result of 94% curing efficiency.^114^ It appears that *K. pneumoniae* bacteriophage could be engineered for CRISPR‐Cas9‐mediated gene targeting with promising therapeutic results. However, before reaching any concrete conclusions, in vitro and in vivo studies are necessary.

### 5.3 *Mycobacterium* spp.

*Mycobacterium tuberculosis* is a single infectious agent causing tuberculosis (TB), which is among the top nine causes of death across the globe. *M. tuberculosis* is a great threat to human society due to its high pathogenesis.^115^ Broadly, the pathogenicity of *M. tuberculosis* is based on reprograming host macrophages that help them to evade elimination, formation of granulomas which assist pathogen survival and lastly conversion of *M. tuberculosis* to a dormant state that resists host defense mechanisms.^115^ In addition, various nontuberculous mycobacterial (NTM) species have emerged as another challenge because the disease caused by them shares clinicoradiological features with TB, Nocardia, and numerous fungal diseases, often resulting in delayed diagnosis.^116^, ^117^ Furthermore, mismanagement of antibiotics and conversion of a susceptible strain into a resistant strain by acquisition of a new antibiotics‐resistance gene through horizontal gene transfer has resulted in the emergence of drug‐resistant *Mycobacterium* strains.^116^, ^117^ There is an urgent need to overcome this problem.

As a recent development in gene editing technology, CRISPR‐Cas has the potential to neutralize antibiotic‐resistant genes in a specific targeted MDR bacterial population or kill them without affecting the beneficial wild‐type populations.^91^, ^95^, ^118^, ^119^, ^120^ A phage‐based delivery system has been described that utilizes an endogenous type III‐A CRISPR‐Cas system of *M. tuberculosis* to target *rpoB* genes of the same organism. The phage could be engineered to deliver mini‐CRISPRs, which are DNA sequences compatible with endogenous CRISPR‐Cas systems, for processing the mini‐CRISPR into crRNA to target the desired gene.^121^ Such systems are expected to specifically and efficiently kill *M. tuberculosis* cells. In another report, the CRISPR associated proteins1 (Cas1) from the *Mycobacterium* type III‐A CRISPR family has been highlighted as a potential candidate for *M. tuberculosis* treatment. The study reported the presence of disrupted Cas1 protein in 57.14% of clinical isolates. Further investigation identified the role of Cas1 in increasing the sensitivity of pathogens against anti‐tuberculosis drugs during drug treatment.^122^ A two‐plasmid‐based base‐editing system has recently been developed with the aim of better understanding the genes and pathways involved in *M. tuberculosis* physiology and antibiotic resistant mechanisms. The system encompasses RecX, to suppress RecA‐dependent DNA repair system; NucS_E107A_, to suppress NucS dependent DNA repair system; the Cas9 nickase fusion protein of cytidine deaminase, and uracil DNA glycosylase inhibitor for efficient base pair conversion in the *M. tuberculosis* genome.^123^ In addition, a genome‐editing Cas9‐based toolbox for site‐specific gene deletion, double mutations, large‐scale genetic mutation, and frameshift mutation in *M. tuberculosis* has been constructed.^124^, ^125^

The potential of using bacteriophages as a therapeutic tool has been evaluated for other *Mycobacterium* infections as well. As per the report, a patient suffering from cystic fibrosis due to *M. abscessus* showed clinical improvement when using genetically modified bacteriophages as therapeutic agents.^126^ Muddy, BPs33ΔHTH‐HRM10, and ZoeJΔ45 were the phages used for the therapy. The latter two are genetically engineered variants of BPs and ZoeJ, which enabled their lytic potentials. Interestingly, no *M. abscessus* was detected from the sputum or serum of the patient after phage injection, and no adverse effect of phage treatment was reported. However, even though the treatment showed promising results, the study should be expanded to similar patients to develop a more thorough understanding. Recently, the lytic phages based therapeutic intervention have been used to treat 20 patients suffering from nontuberculous *Mycobacterium* infection. No adverse reactions were reported in any of the patients treated with the phages and 11 patients displayed a favorable clinical outcome. Antibodies against phages were reported in few of the patients that were intravenously injected with phages, however no phage resistance was reported in 11 patients treated.^127^ In addition, many studies have utilized CRISPR‐assisted phage engineering to prevent conventional antibiotic drug resistance in several bacteria.^128^, ^129^, ^130^, ^131^ Furthermore, advancements in biotechnology motivate further modification of phage particles for improving a variety of features, such as improving the ability to penetrate biofilm‐forming bacteria, creating more specific and stable phages, increasing phage efficacy, and extending the spectrum of phage lytic activities.^132^, ^133^, ^134^ These modifications in phages using the CRISPR‐Cas system or using phage for CRISPR‐Cas delivery to target antibiotic resistant or essential genes could be beneficial for combating different mycobacterial‐, NTM‐, as well as MDR‐ or extensively drug‐resistant strains. In summary, CRISPR‐Cas tools for *Mycobacterium* genome editing have been used in several studies. However, the use of this in *M. tuberculosis* for resensitization against antibiotics, target‐specific killing, or phage‐mediated CRISPR‐Cas delivery is yet to be explored.

### 5.4 *Salmonella* spp.

Worldwide, salmonellosis is a very common foodborne disease. It has been associated with outbreaks in several countries, resulting in high morbidity and mortality.^135^, ^136^, ^137^, ^138^ Seeking to curb salmonella infections, Nikkhahi et al. discovered a lytic bacteriophage and demonstrated its therapeutic value in mice.^139^ Oral administration of 2 × 10^9^ plaque‐forming units/mouse was very effective in protecting the mouse against *Salmonella* infection. The study suggested that the isolated bacteriophage could be a potential candidate for therapeutic purposes and might help prevent foodborne illnesses. A study has demonstrated that the phage PA13076 protects mice from a lethal dose of *S. enteritidis* 13076 by reducing the concentration of bacterial cells in blood and various organs such as the intestine, liver, spleen, and kidney.^140^ The results suggest that phage PA13076 has a remarkable potential to treat *S. enteritidis* infections. Another study has comprehensively analyzed the phage susceptibility variation in the two strains of *S. enterica* serovar Typhimurium DT104 and DT104b.^141^ These finding could be helpful in terms of understanding the host–phage interaction and might encourage the development of CRISPR‐assisted phase‐based treatment for salmonellosis and related infections.

### 5.5 Staphylococcus aureus

*S. aureus* is a Gram‐positive pathogenic bacterium that is highly resistant to antibiotics. Methicillin‐resistant *S. aureus* (MRSA) is the prime culprit of global *S. aureus* bacteremia and causes metastatic or other infections such as endocarditis and sepsis.^142^ The mortality rate for systemic infection due to MRSA is greater than 50%.^143^ In addition, as per a CDC report, MRSA alone was responsible for 80,000 infections and 11,285 deaths in 2011.^144^ Furthermore, as per a recent meta‐analysis of patients with positive *S. aureus* infection from MEDLINE, Cochrane Database of Systematic Reviews and Embase databases (1991 to May 2021), one in every four patients dies within 3 months due to *S. aureus* bacteremia.^145^ Considering the previously mentioned statistics, along with the fact that MRSA is resistant to β‐lactam, quinolones, aminoglycosides, and macrolides, the pathogens pose a threat to global health.^143^ Thus, researchers are investing time and effort into discovering novel classes of antibiotics and alternatives to treat MDR *S. aureus* infections. As an alternative to antibiotics, phages have shown potential to treat *S. aureus* infections. Recently, a clinical trial has shown significant reductions in the staphylococci without any adverse effects after intravenous administration of *Myoviridae* bacteriophages (AB‐SA01) in patients.^146^ In 2019, the Food and Drug Administration (FDA) approved phase I/II clinical trials of phages for treatment of ventricular‐assisted device infections.^147^ In another study, bacteriophage ΦNM1 was used to deliver the CRISPR‐Cas9 expressing phagemid to target aminoglycoside phosphotransferase (*Aph‐3*) and penicillin binding protein (*mecA*) genes in drug‐resistant isolates of *S. aureus*.^91^ The system could successfully resensitize kanamycin‐resistant or methicillin‐ and tetracycline‐resistant *S. aureus* strains and inhibit their growth. The same system also reported promising results when ΦNM1‐carrying constructs were used to treat *S. aureus* skin infectioremn in a CD‐1 mouse model.^91^ Likewise, a CRISPR‐Cas9 system programmed to target micrococcal nuclease (*nuc*) and type VII secretion system extracellular protein A (*esxA*) was integrated into the genome of a φSaBov temperate phage. In the study, *S. aureus* ST1, ST5, ST8, and ST36 and CTH96 were used to test the potential of the engineered phages as therapeutics.^148^ The report highlights a significant reduction of *Staphylococcus* (1 × 10^5^ CFU) due to engineered phages compared to a native unmodified phage treatment (1 × 10^9^ CFU). This study was extended in an in vivo C57BL/6 mouse model to treat skin infection. Remarkably, the study demonstrated a significant bacteriophage bactericidal activity in both in vitro and in vivo.^148^ Moreover, the CRISPR‐Cas9‐modified temperate phage has also been used to investigate the treatment of osteomyelitis (bone infection) and soft tissue infection in Sprague Dawley female rats, which is often caused by antibiotic‐resistant *S. aureus*. The study focused on targeting the *nuc* gene of *S. aureus* ATCC 6538‐GFP by integrating a programmed CRISPR‐Cas9 system into a noncoding genomic region of φSaBov phage. The engineered phage was also challenged in vitro to treat *S. aureus* biofilm. Intriguingly, in vitro qualitative fluorescent imaging showed significant anti‐biofilm activity compared to fosfomycin and vancomycin antibiotics, whereas quantitative anti‐biofilm effects gradually increased over time for phage, fosfomycin, and phage‐fosfomycin treatments delivered via alginate hydrogel.^149^ In addition, the engineered phage delivered via hydrogel enabled reduction of soft tissue infection in the rat model but failed to do the same for bone infections.^149^ In another study, bactericidal activity of M13 phage loaded with a vector that facilitated Cas13a expression to the target penicillin binding protein (*mecA*) of *S. aureus* USA300 was demonstrated. The study highlighted the superior bactericidal activity of Cas13a compared to Cas9 nuclease. Furthermore, the proposed system demonstrated remarkable therapeutic effects in a *G. mellonella* model.^94^ In summary, phages to treat *S. aureus* infections have entered into clinical trials, and notably phages carrying a CRISPR‐Cas system have shown potential as bactericidal agents for resensitization and eradication of *S. aureus* infections. Nevertheless, there are a few shortcomings that need to be addressed before applying phage‐based CRISPR‐Cas to combat *S. aureus* infections such as generalized transduction of virulent genes and the narrow host range. However, genetic engineering resources are emerging^148^ that might allow safe and effective therapeutics to provide a path to clinical trials.

## 6 ADAPTIVE LABORATORY EVOLUTION FOR ENHANCING PHAGE FITNESS

Phage stability and effectiveness are the major factors for proficient phage therapy. Natural phages are sensitive to several environmental factors such as temperature, solute present in the sample, and ultraviolet (UV) light. A simple solution to overcome this hurdle could be adaptive laboratory evolution, a method to improve the evolutionary fitness and adaptability of organisms in changing environments.^150^ The method uses mutagenesis and selective environments to challenge and drive the strains to adapt well in desired growth conditions.^151^ In one study, three wild‐type phages, Wc4 belonging to the *Myoviridae* family and two phages CX5 and P‐PSG‐11 of the *Podoviridae* family, were used for adaptive laboratory evolution to improve their stability at elevated temperature.^150^ The phages were treated at 60 °C for 5 cycles, and they displayed greater stability when exposed to 60 °C for 1 h after storage at 37 °C for 60 days. The lytic efficiency and infectivity of the adapted phage were unaltered throughout the evolution process.^150^ Whole genome sequencing data revealed beneficial single substitutions in phage tail tubular proteins that enabled phages to tolerate higher temperatures. This result provided new insight into the stability of adapted phages at higher temperature for easier transportation and storage.^150^ In another study, the T7 phage was exposed to 30 cycles of lethal UV light for selection of a phage with improved UV resistance. The results showed that, while the UV exposure killed 99.99% of wild‐type phages, the adapted phage had 50‐fold improved UV light resistance and exhibited improved robustness and stability.^144^ As per the report, a 2.1‐kb deletion and three substitutions in the early and structural gene, respectively, were found in most of the adapted phages and could be the reason for improved fitness against UV light.^144^ In a recent study, a method known as chemically accelerated viral evolution (CAVE) was developed to enhance the evolution of desired characteristics in bacteriophages. To drive bacteriophage evolution to the desired phenotype, CAVE uses an iterative round of mutagenesis, which is coupled with selection criteria. Briefly, CAVE involves four steps: (i) introduction of mutations across the phage genome, (ii) host infection to generate a pool of mutant phage, (iii) application of selection criteria, and (iv) analysis of phage variants and cycle repetition. In the same study, CAVE was successfully and efficiently tested to improve the thermal stability of T7 bacteriophages.^152^ In brief, adaptive laboratory evolution is a flexible technique that could be applied to improve the stability of a promising therapeutic phage for better storage, transportation, and therapy.

## 7 DIRECTED EVOLUTION TO IMPROVE PHAGE THERAPY

Directed evolution is a method mimicking the natural selection process for genes and their corresponding proteins toward a user‐defined goal. Directed laboratory evolution is similar to adaptive laboratory evolution; however, the aim in directed laboratory evolution is to drive the protein toward improved functionality.^151^, ^153^, ^154^ The concept of directed laboratory evolution has been used to improve the infectivity and host specificity of phages for therapeutic purposes. In one such study, mycobacteriophage (ATCC® 11759B1TM) that infects a nonpathogenic strain of *Mycobacterium*, namely, *M. smegmatis* was used for directed evolutionary studies. In the study, directed evolution was used as a tool for increasing the lytic activity and infectivity of mycobacteriophage. The study investigated the effect of phage inoculum size to achieve desired adaption.^155^ Interestingly, their data suggest that using a smaller phage inoculum during evolution studies helps to achieve higher titer, greater plaque size, and efficient lysis compared to larger regimes. As some mycobacteriophage can infect both *M. smegmatis* and *M. tuberculosis*, the same study could be expanded using *M. tuberculosis* as a host for enhancing the potential of phage as a therapeutic.^155^ In another study, directed evolution was used for limiting the host range of T7 bacteriophages. T7 phage was grown in the presence of five restrictive and one permissive strain for propagation. The restrictive strain was a *trxA* mutant, where *trxA* encodes thioredoxin, which is an essential subunit for phage DNA polymerase. This setup allowed directed evolution of the phage for narrowing the host range by recognizing specific forms of LPS present on the cells while avoiding the others. The resulting evolved phage had mutations on tail genes 11 and 12 and on tail fiber gene 17 that altered their specificity. The experimental set up used in this study could be channeled for bacteriophage‐mediated elimination of bacterial serotypes in a mixed population.^156^ In a recent study, laboratory evolution was used on bacteriophage λ as a proof of concept to encounter phage‐resistance of *E. coli* B strain REL606 for better therapeutic applications. Phages were trained for 28 days and were able to suppress the bacteria with 1000‐fold higher efficiency for 3–8 times longer compared to their ancestor strain. Intriguingly, it only took one mutation step of the bacteria to become resistant to the untrained phage, whereas it took multiple mutations in bacteria to achieve the same for laboratory evolved phages.^157^ Thus, directed evolution is a promising strategy to increase the therapeutic value and specificity of the phage, and the possible next step could be using the evolved phage against clinical isolates and to test the therapeutic potential of the evolved phage in in vivo models.

## 8 STRATEGIES TO DELIVER THERAPEUTIC PHAGES

Bacteriophages have immense potential as antibacterial candidates in the upcoming post antibiotic era, as is evidented by in vitro as well as clinical studies. It is crucial to ensure that the bacteriophages are delivered at the site of infection for the treatment to be effective, failing to which the treatment could be ineffective.^158^ This could be challenging, especially with therapeutic phages, as they face difficulty in penetrating tissues.^159^ In addition, if phages are delivered without preparation, they are more likely to be degraded by enzymes or change with pH.^160^ They are also at high risk of being inactivated by the host immune system.^159^, ^161^ To overcome this hurdle, much research is being focused on devising delivery strategies that could allow bacteriophages to reach to the target site and exhibit their full potential. Many of the strategies involve encapsulating or entrapping the phages within liposomes, fibers, and hydrogels.^159^, ^162^, ^163^, ^164^

Liposomes are enclosed lipid bi‐layered nanostructures, spherical in shape, and hollow, allowing them to carry aqueous solutions. Bacteriophages enclosed by liposomes have been shown to protect the phages against host environmental insults such as acidic pH and degrading enzymes found in the stomach and gut, respectively.^159^ In one such study, phages against *Salmonella* that were encapsulated by cationic liposomes protected them against simulated gastric fluid (SGF) of pH 2.8. In addition, encapsulation has been shown to improve the retention of phages in chicken intestinal tracts.^165^ In another study, the potential of bacteriophages loaded in cationic liposomes was observed to escape the host immune system. Interestingly, it has been observed that cationic liposomes loaded with bacteriophages offer them 100% protection against anti‐phage antibodies of mice, whereas the phages without encapsulation were neutralized within 3 h of reaction.^166^

In addition to stability, liposomes have also been investigated for their therapeutic value in different in vivo models. For instance, enhanced efficacy of encapsulated bacteriophages is observed compared to freely delivered phages for protecting broilers against *Salmonella* spp.^165^ Similarly, the therapeutic effects of freely delivered phages compared to liposomal encapsulated bacteriophage against *K. pneumoniae* using burn wound mice models have been examined. Higher reduction of bacterial load was reported in blood and other organs of mice when treated with encapsulated phages compared to phage delivered freely. In addition, encapsulated phages offered higher retention values and greater specificity to cure the infection. Moreover, phages delivered through liposomal preparation protected the mice from death even if the treatment was delayed for 24 h.^167^ Similar results were observed for wound healing in a diabetic mouse model having *S. aureus* infection.^168^ The caliber of bacteriophages encapsulated in the cationic liposome has also been evaluated for targeting pathogens residing inside the cells. In one such study, liposome encapsulated bacteriophages were able to clear 94.6% of *K. pneumoniae* residing in the macrophage, and this result was in contrast to free bacteriophages that were unable to penetrate eukaryotic cells.^166^ Other than assisting elimination of intracellular pathogens, liposomal bacteriophages also help to eradicate biofilms more efficiently. In one study, a synergistic effect of using bacteriophages along with antibiotics was examined against *K. pneumoniae* biofilms. Using the clinical achievable antibiotic concentration increased the efficacy of the liposomal encapsulated phage for reducing the bacterial load in young as well as mature biofilms. In the same study, the synergistic effect for reduction of bacterial load via free bacteriophages was not significant in mature biofilms.^166^ Even though liposomal encapsulation offers an excellent solution to bacteriophage storage and stability while increasing therapeutic value, preparing such encapsulations is a challenge. For example, smaller liposomes have longer retention time in the body and a better chance to be taken up by the cells to target the intracellular pathogen. However, it is difficult to control the sizes of the liposomes, and the encapsulation yield of phages inside them is lower. Techniques such as microfluidic mixing have been devised to overcome the issue but work only for certain types of phages while others seem to aggregate or attach to the surfaces of the liposome.^159^

Hydrogel can be defined as a 3D cross‐linked network of hydrophilic polymers with the capacity to retain a large amount of water.^169^ Like liposomes, hydrogels have been examined for their roles in stability, storage, and therapeutic effects of phages. For example, the use of alginate was investigated for *S. aureus* phage K stability in SGF of pH 2.5. The encapsulation via alginate hydrogel microspheres improved the phage stability compared to that of free phages in SGF, and these microspheres further improved phage survival when calcium carbonate microparticles were included in the formulation.^170^ Similarly, Chitosan‐alginate beads along with a honey and gelatin matrix for encapsulation have been investigated for protecting *E. coli* bacteriophage ZSEC5 against acid stress. The same encapsulation has been highlighted to prevent phage degradation at elevated temperature.^171^ Likewise, alginate/CaCO_3_ encapsulated cocktails of three phages against *Salmonella* were tested for their ability to be orally administered in chickens. The alginate/CaCO_3_ preparation allowed 100% encapsulation of phage cocktail, were stable in the stomach, and retained in the intestines during in vivo studies. A high antibacterial activity of encapsulated phage cocktail was reported against *Salmonella* infections.^172^ The therapeutic effect of hydrogel gel encapsulated phages have also been investigated in rat models for *S. aureus* mediated osteomyelitis and soft tissue infection. As per the study, alginate encapsulated phages gave therapeutic effect comparable to high dose of fosfomycin for skin infection models, however the same was not effective for rat models with bone infections.^149^

Hydrogels have also been used to devise smart systems based on pH responsive surface coatings for long term catheters that release phages during infection. At the time of infection, pathogens such as *Proteus mirabilis* colonize and form a biofilm that results in an increase in the pH of the surrounding area. The increase in pH acts as a stimulus for the “trigger layer” that leads to release of the phage from the lower “reservoir layer” of the hydrogel. In a study of an in vitro bladder model, phages entrapped in a pH‐responsive hydrogel made from poly(methylmethacrylate‐co‐methacrylic acid) delayed blocking of the catheter due to biofilm formation.^173^ In addition to the pH responsive surface coating hydrogel‐based smart systems, thermo‐responsive polymer‐based smart systems have been designed for wound infections. These thermo‐responsive polymers remain intact at low temperature, but the polymers dissolve with an increase in temperature, as is often observed during a bacterial skin infection.^174^ If the phages are entrapped in a thermo responsive polymer, there would be a gradual release of the phages during infection.^174^ A gel matrix of allylamine copolymerized with poly‐*N*‐isopropyl‐acrylamide is an example of a thermo‐responsive polymer used as nanospheres to entrap *S. aureus* phage K and added to a nonwoven fabric for usage in adhesive bandages.^174^ Hydrogels are undergoing rapid development. One of the major limitations of traditional hydrogels involves degradation and mechanical properties, although new hydrogel formulations are steadily improving.^175^ Also, stabilization of phages during delivery is important because it correlates with the success rate of the therapy. Incorporation of phage after gel preparation increases the chances for phage stability. In addition, the viability of a phage would change with a change in hydrogel formulation.^176^ Thus, there is need for a study focusing on long‐term stability and safety based on hydrogel and phage combinations.

Apart from the liposomes and hydrogels, phages bound to fibers have also been studied for their therapeutic effects. Phages immobilized on fibers are a simplistic yet effective strategy for topical administration of phages during wound dressing or in bandages.^159^ The nanofibers are produced via electrospinning, and the bacteriophages are added to liquid polymer prior to the electrospinning process. Thus, during the production of nanofibers, the phage is encapsulated in the fiber and confer antibacterial trait to the resulting product. Commercially available phage cocktails such as Fersis and PhageStaph have been immobilized on nanofibers formed by polyethylene glycol and polyester urea. The resulting phage immobilized on nanofibers displayed antimicrobial activity to their respective hosts until 80 h of exposure.^177^ A phage‐based washable and nontoxic wound dressing system has also been devised using polycaprolactone nanofibers to covalently immobilize *Pseudomonas* bacteriophage on its surface. The resulting biomaterial was effective until 25 cycles of washing.^159^ In another study, the T7 phage loading efficiency, its distribution, and release from the cellulose microfiber using electrostatic interactions, nonspecific adsorption, and protein–ligand binding as immobilization approaches were investigated. Electrostatic interactions demonstrated 15%–25% phage loadings normalized to the initial titer of the phage, whereas the system for nonspecific adsorption and protein–ligand interaction was not significant. Furthermore, slow release of the phage was documented from cellulose microfibers when phages were attached using electrostatic interactions as an immobilization strategy.^178^ One major challenge for encapsulating phage on fibers is its stability. During the electrospinning process, the polymer and phage are exposed to high voltage, resulting in rapid evaporation of water and changes in osmotic state, which leads to drying of the phage and low viability during storage. However, addition of magnesium salts and excipients such as trehalose has been observed to improve the viability of the phage during electrospinning process and storage.^177^

The recent increase in number of clinical trials using intravenous or oral phages provides an evidence of growing global interest in phage therapy. However, large‐scale utilization of phages in clinical settings are limited by various factors such as need for repeated administration, narrow host range and loss of activity in physiological condition. Another limitation is in vivo decay of bacteriophage lytic activity by physiological factors such as change in pH or serum inactivation. Therefore, sustained release of phages from biomaterials, which are locally implanted at the site of infection, might prolong its residing time for better treatment and improve its therapeutic efficacy.^158^, ^162^ Additionally, phage preparation is a challenging task and needs to be addressed to successfully bring this therapy into clinical practice. Phages are biological entities that are relatively unstable compared to other chemical products. There is need for formulations that maintain phage viability during preparation, remain intact during storage, and are robust in host environmental conditions. Although this has been the focus of many studies, along with the growing list of encapsulating materials for phage, phage preparation could be a strong asset for successful intervention in the healthcare system.

## 9 CONCLUSION

With continued excessive use of antibiotics, human society is approaching a post‐antibiotic era where the antibiotics are turning ineffective. Immediate action is needed or a common infection or minor injury could prove to be fatal. Phage therapy could be an alternative approach for controlling rapidly evolving MDR pathogens that are difficult to treat with existing antibiotics. Diversity and adaptability are advantages to using phages to treat an infection. Furthermore, literature shows successful use of phages as therapeutics. With the growing genome engineering tools and techniques and increasing knowledge of phage biology, it is possible to engineer a phage with desired characteristics. These techniques and tools have enabled an increase in the overall fitness of the phage, significantly influencing its therapeutic ability. Furthermore, integrating CRISPR‐Cas technology into the phage has shown potential for target‐specific removal of pathogens from a mixed population. The therapeutic effect of this technology could be different from the currently available broad‐spectrum antibiotics. One of the drawbacks of broad‐spectrum antibiotics is the spread of resistant genes across bacterial species, and alteration of the host microbiome. If the pathogen behind infectious diseases is known, CRISPR‐assisted phage therapy could help to overcome some of these issues. CRISPR‐Cas has also enabled strategies such as PRESA, which has shown promise as compared to lytic phages for therapeutics. The use of phage acquired CRISPR could alter the global genetic landscape of the bacterial population. However, a large proportion of this change relates to antimicrobial‐resistant genes of the targeted pathogens, which is also the need considering the current global crisis. For successful bacteriophage therapy, encapsulation of a phage is necessary for stabilization during storage and treatment. Encapsulation has also enabled researchers to construct smart phage release strategies. Over the years, much attention has been given to formulating various recipes for phage encapsulation, and this could be key for its clinical success. In summary, phages integrated with a programmable endonuclease are a promising therapeutic candidate to combat MDR pathogens. However, rigorous assessment and data from clinical trials using genetically engineered or evolved phage as therapeutics are lacking. Until data regarding safety are gathered, phages, whether wild type, laboratory evolved, or genetically engineered, could be the last option when other treatments fail.

## AUTHOR CONTRIBUTIONS

**Khushal Khambhati:** Writing – original draft (lead). **Gargi Bhattacharjee:** Funding acquisition (equal); writing – original draft (supporting). **Nisarg Gohil:** Funding acquisition (equal); writing – original draft (supporting). **Gurneet K. Dhanoa:** Writing – original draft (supporting). **Antonia P. Sagona:** Conceptualization (equal); writing – review and editing (supporting). **Indra Mani:** Writing – original draft (supporting). **Nhat Le Bui:** Writing – original draft (supporting). **Dinh‐Toi Chu:** Conceptualization (equal); writing – review and editing (equal). **Janardhan Keshav Karapurkar:** Writing – original draft (supporting). **Su Hwa Jang:** Writing – original draft (supporting). **Hee Yong Chung:** Supervision (supporting); writing – review and editing (equal). **Rupesh Maurya:** Writing – original draft (supporting). **Khalid J. Alzahrani:** Funding acquisition (equal); writing – review and editing (equal). **Suresh Ramakrishna:** Conceptualization (equal); funding acquisition (equal); supervision (lead); writing – review and editing (supporting). **Vijai Singh:** Conceptualization (equal); funding acquisition (equal); supervision (lead); writing – review and editing (equal).

## CONFLICT OF INTEREST

The authors have no competing interests to declare.

### PEER REVIEW

The peer review history for this article is available at https://publons.com/publon/10.1002/btm2.10381.

## Data Availability

Data sharing not applicable to this article as no datasets were generated or analyzed during the current study.
